# Rethinking viral evolution: How BIAS mechanisms and Gamma-Poisson overdispersion redefine lethal mutagenesis

**DOI:** 10.3389/fmicb.2026.1819980

**Published:** 2026-05-13

**Authors:** Deya Wang, Xianping Zhang, Qingle Chang

**Affiliations:** College of Life Sciences, Zaozhuang University, Zaozhuang, China

**Keywords:** BIAS mechanism, Gamma-Poisson distribution, lethal mutagenesis, RNA virus, viral evolution

## Introduction

Viruses are known to incur large numbers of errors during replication, yet most still manage to proliferate and spread among hosts. Lethal mutagenesis is based on the idea that antiviral drugs can raise viral error rates above a tolerable threshold, causing descendant genome copies to accumulate too many errors to sustain productive infection (Bull et al., 2007; Antoneli et al., 2012). Traditional thinking has treated this process as a mean-field phenomenon, assuming that descendant genome copies incur similar numbers of errors and thus follow a Poisson-like distribution. Emerging data suggest that this assumption may not hold true. Instead, individual genome copies of the same virus may incur very different numbers of errors, producing an overdispersed pattern better captured by Gamma-Poisson (negative binomial) dynamics (Arcos and Lauring, 2026). We propose that, in certain viral systems, this pattern reflects biologically structured mutation-rate heterogeneity rather than stochastic noise. We further suggest that the Bottleneck, Isolate, Amplify, Select (BIAS) framework may provide a mechanistic basis for this variance structure (Perdoncini Carvalho et al., 2022). Successful viral replication within a host cell may require the cooperation of numerous coexisting genome copies, even though replication itself remains strongly bottlenecked. Under such conditions, many genome copies may help support replication, but only a very small and stochastic subset escapes the reproductive bottleneck to found descendant lineages. If these bottlenecks are reinforced by intracellular segregation, error burdens need not be evenly distributed among descendant genomes, and tissue-level sampling would be expected to recover an overdispersed pattern. At the same time, bottleneck-enforcing proteins (BNEPs) may limit complementation, thereby facilitating the persistence of relatively high-fidelity lineages and maintaining a protected low-mutation “long tail” within the population. Such a variance structure may raise the effective extinction threshold and prolong time to extinction, helping to explain why mutagenic antivirals can reduce viral load without necessarily achieving sterilizing extinction. We emphasize that this framework is conditional rather than universal, and is most relevant where genome mixing is restricted and complementation is limited. Consequently, “anti-variance” interventions may offer a useful therapeutic strategy for making viral populations more predictably susceptible to extinction.

## Main text

The fact that lethal mutagenesis therapies, such as molnupiravir against SARS-CoV-2, often fail to achieve complete sterilizing extinction suggests that current models based on Poisson-distributed mutation counts may underestimate the mutational burden required for extinction in some viral systems (Sanderson et al., 2023; Crotty et al., 2001; Fischer et al., 2022; Shannon et al., 2020; Standing et al., 2024). One possible explanation is that these models do not sufficiently account for mutation-rate heterogeneity within viral populations (Arcos and Lauring, 2026; Allman et al., 2022; Sardanyés et al., 2024). Emerging data and theoretical work suggest that viral mutation rates are not constant, but variable, and that Gamma-Poisson distributions may better capture the overdispersed nature of viral mutation counts, in which the variance exceeds the mean (Arcos and Lauring, 2026). This variability may not simply reflect stochastic noise. Rather, in some contexts, it may reflect biologically structured heterogeneity that enhances the resilience of viral populations to mutagenic treatment. At the same time, this interpretation should be considered alongside virus-specific biological features. For example, coronaviruses encode a proofreading exonuclease that lowers baseline mutation rates and complicates direct comparison with many other RNA viruses (Gribble et al., 2021; Smith et al., 2013; Li et al., 2025; Moeller et al., 2022). In addition, altered replication fidelity has been reported in several human viruses, including HIV, Influenza A virus, poliovirus, herpes simplex virus, and adenovirus, indicating that fidelity modulation is not merely a theoretical possibility, but a biological phenomenon observed across distinct viral systems (Preston et al., 1988; Maldonado and Mansky, 2018; Lin et al., 2019; Kawasaki et al., 2023; Cheung et al., 2014; Vignuzzi et al., 2008; Pfeiffer and Kirkegaard, 2003; Smith and Denison, 2012; Jiang et al., 2009).

The BIAS (Bottleneck, Isolate, Amplify, Select) hypothesis offers a possible mechanistic framework for understanding this variability (Arcos and Lauring, 2026; Perdoncini Carvalho et al., 2022; Qu et al., 2020). According to this hypothesis, some viruses may impose strong intracellular bottlenecks via bottleneck-enforcing proteins (BNEPs), thereby restricting the number of replicating genomes in each cell to a small and stochastic subset. By isolating genomes within distinct replication complexes, BIAS may allow relatively high-fidelity genomes to persist, whereas more error-prone genomes are more likely to accumulate mutations and eventually be lost from the population (Perdoncini Carvalho et al., 2022; Qu et al., 2024). Under these conditions, intracellular segregation may reduce the effective scope of complementation and favor the persistence of relatively high-fidelity genomes, whereas more error-prone genomes may continue to accumulate mutations and become less competitive (Qu et al., 2024; Leeks et al., 2021). Importantly, this does not mean that complementation is absent across all viral systems. Rather, the relevance of BIAS depends on how strongly intracellular organization, limited genome mixing, or effective privatization of gene products constrains the sharing of public goods.

**Figure 1:**
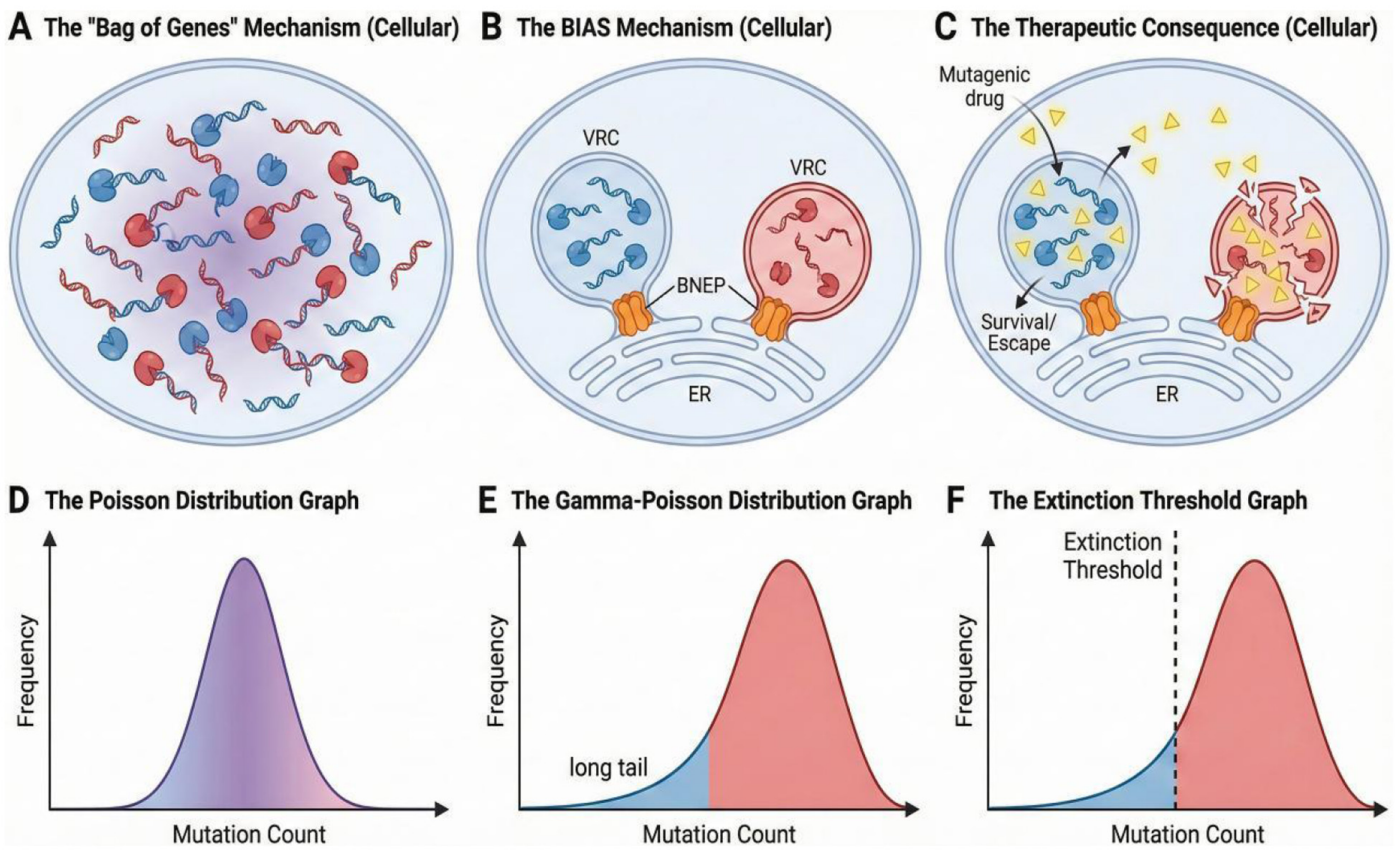
The variance trap: BIAS-driven intracellular isolation may convert polymerase heterogeneity into Gamma-Poisson overdispersion and limit the effectiveness of lethal mutagenesis. **(A)** In a “bag-of-genes” cytoplasm, polymerases and genomes mix and cross-complement, averaging fidelity phenotypes across lineages. **(B)** Under BIAS, bottleneck-enforcing proteins (BNEPs) channel replication into spatially separated viral replication complexes (VRCs), favoring cis-preferential replication and preserving lineage-specific fidelity (blue, high fidelity; red, low fidelity) when mixing is restricted. **(C)** Mutagens increase the mean mutation burden but may preferentially purge low-fidelity compartments, while high-fidelity VRCs may persist and potentially contribute to escape. **(D)** If mutation rates were uniform, mutation counts would approximate a narrow Poisson-like distribution (variance ≈ mean). **(E)** When fidelity heterogeneity preserved by isolation, mutation counts become overdispersed (Gamma-Poisson/negative binomial), yielding a protected low-mutation “high-fidelity” tail (blue) alongside a higher-mutation bulk population (red). **(F)** Following a drug-induced rightward shift in the distribution, most of the bulk population crosses the extinction threshold, whereas the high-fidelity tail may remain below it. This suggests that variance structure raises the effective extinction threshold and prolongs time to extinction relative to Poisson-based expectations.

In this framework, the extinction threshold—the point at which mutation drives a population to extinction—is not fixed, but may vary with mutation-rate heterogeneity (Arcos and Lauring, 2026). If maintained by intracellular segregation, overdispersion may raise the effective extinction threshold and prolong time to extinction, making mutagenic collapse harder to achieve than predicted by uniform-rate models. This suggests that models assuming homogeneous mutation rates may underestimate the treatment intensity required to drive a viral population to extinction. By contrast, Gamma-Poisson models suggest that the protective “long tail” of high-fidelity variants, preserved by BIAS-mediated isolation, may be more resilient to mutagenic drugs than previously appreciated (Figure 1).

Moreover, the BIAS hypothesis suggests that viruses may evolve mechanisms that preserve genome-level isolation (Qu et al., 2020, 2024). A virus that cannot maintain sufficient segregation may become more vulnerable to cross-complementation, whereby defective genomes can borrow functional products from others, weakening selection for replication fidelity. At the same time, this reasoning is unlikely to apply equally across all viruses, because many viral systems show substantial public-goods sharing, complementation, and genome mixing (Leeks et al., 2021; Meir et al., 2020). We therefore do not regard BIAS as a universal rule, but as a conditional framework that is most relevant to viral systems with strong bottlenecks, limited complementation, marked superinfection exclusion, reduced sharing or effective privatization of gene products, and replication in structurally or functionally segregated compartments. This framework may be particularly relevant to some positive-strand RNA viruses, including plant viruses such as *turnip crinkle virus* (TCV) and *tobacco mosaic virus* (TMV), as well as animal viruses such as *hepatitis C virus* (HCV) and *west nile virus* (WNV). Although these systems may not fit every aspect of the BIAS framework equally well, they still show how superinfection exclusion and spatially organized replication can reduce genome mixing and limit broad sharing among coexisting genomes (Qu et al., 2020, 2024; Zou et al., 2009; Webster et al., 2013). Its applicability is therefore likely to depend on how extensively intracellular isolation is maintained in a given viral system. Within viral populations that retain a resilient tail of high-fidelity variants, mutagenic drugs such as molnupiravir may be less effective than Poisson-based models predict, because this low-mutation subpopulation may remain below the extinction threshold despite treatment.
